# Electrospun Benzimidazole-Based Polyimide Membrane for Supercapacitor Applications

**DOI:** 10.3390/membranes12100961

**Published:** 2022-09-30

**Authors:** Yu-Hsiang Lu, Yen-Zen Wang, Ming-Ying Tsai, Hong-Ping Lin, Chun-Han Hsu

**Affiliations:** 1 Department of Chemical and Materials Engineering, National Yunlin University of Science and Technology, No. 123, Sec. 3, University Road, Douliou 64002, Taiwan; 2 Department of Chemistry, National Cheng Kung University, No. 1 University Road, Tainan City 70101, Taiwan; 3 General Education Center, National Tainan Junior College of Nursing, No. 78, Sec. 2, Minzu Road, Tainan City 700, Taiwan

**Keywords:** supercapacitor, separator, polyimide, electrospin, benzimidazole

## Abstract

A benzimidazole-containing diamine monomer was prepared via a simple one-step synthesis process. A two-step procedure involving polycondensation in the presence of aromatic dianhydrides (4,4′-oxydiphthalic anhydride, ODPA) followed by thermal imidization was then performed to prepare a benzimidazole-based polyimide (BI-PI). BI-PI membranes were fabricated using an electrospinning technique and were hot pressed for 30 min at 200 °C under a pressure of 50 kgf /cm^2^. Finally, the hot-pressed membranes were assembled into supercapacitors, utilizing high-porosity-activated water chestnut shell biochar as the active material. The TGA results showed that the BI-PI polymer produced in the two-step synthesis process had a high thermal stability (T_d5%_ = 527 °C). Moreover, the hot-press process reduced the pore size in the BI-PI membrane and improved the pore-size uniformity. The hot-press procedure additionally improved the mechanical properties of the BI-PI membrane, resulting in a high tensile modulus of 783 MPa and a tensile strength of 34.8 MPa. The cyclic voltammetry test results showed that the membrane had a specific capacitance of 121 F/g and a capacitance retention of 77%. By contrast, a commercial cellulose separator showed a specific capacitance value of 107 F/g and a capacitance retention of 49% under the same scanning conditions. Finally, the membrane showed both a small equivalent series resistance (R_s_) and a small interfacial resistance (R_ct_). Overall, the results showed that the BI-PI membrane has significant potential as a separator for high-performance supercapacitor applications.

## 1. Introduction

Supercapacitors are a clean-energy storage system with a higher power density and longer cycle life than conventional rechargeable batteries [1,2,3]. Compared with conventional dielectric capacitors, supercapacitors can store significantly more energy due to their porous electrodes, which offer a greater surface area for ion adsorption and desorption [4,5]. The electrolyte solutions used in supercapacitors may be either aqueous or organic. Of the two types of electrolytes, organic electrolytes have a wider potential window (>1.5 V) [6,7,8], and thus have a better energy storage performance.

One of the most important components in supercapacitors and other energy storage systems is the membrane, which physically separates the anode and cathode and prevents internal short circuits between them. The main requirements for a membrane include high ionic conductivity, good chemical and mechanical properties, and high thermal stability [9,10,11,12]. The literature contains many studies on the fabrication of functionalized separators capable of meeting these requirements. For example, Berrada et al. [13] fabricated tough copolymer polyimide (PI) membranes with a tensile modulus of 2.4 GPa and a tensile strength of 95 MPa. Liu et al. [14] synthesized membranes with an improved modulus and tensile strength of 5.7 GPa and 180 MPa, respectively, achieved through a closer packing of the polymer chains. Hu et al. [15] prepared polyimides-based separators using an electrospinning method. The separators not only had a high mechanical strength and thermal stability but also a high ionic conductivity in electrochemistry applications due to their high porosity. 

Polyimides (PIs) have been extensively studied due to their excellent mechanical and electrical properties and superior chemical and thermal stability [13,14,15,16,17]. PIs have been widely used to fabricate many different membranes, fibers, coatings and adhesives [16,17,18,19]. However, to meet the requirements of emerging high-performance supercapacitors and other clean-energy storage systems. PIs with even better properties, particularly higher porosity and superior thermal and dimensional stability, are urgently required [20]. The favorable properties of PIs stem mainly from the ring structures of benzoxazole, furan, pyrimidine and pyridine in the polymeric backbone [21,22]. Among these structures, aromatic polybenzimidazoles (PBIs) are particularly attractive on account of their outstanding mechanical and dielectric properties at high temperatures [23,24]. Several studies have attempted to realize high-performance separators for supercapacitor applications by combining the advantages of PIs and PBIs, respectively, via a physical blending process [25,26]. However, the resulting materials have only partial miscibility due to the inherent stiffness of the polymer chains and the competitive nature of the intermolecular interactions between them. Consequently, the feasibility for preparing homogeneous materials with equivalent properties through the copolymerization of monomers has attracted growing interest in recent years. Xu et al. [27] synthesized a symmetric diamine monomer containing benzimidazole rings (6,6′-bis[2-(4-aminophenyl) benzimidazole] (BAPBI)) through a benzimidazolization condensation method and then obtained corresponding PIs via a two-step polymerization process. The PIs were shown to have a high thermal stability and an outstanding mechanical performance as a result of their rigid-rod structures and the inclusion of hydrogen bonds between the macromolecular chains. 

In the present study, BAPBI is synthesized in a one-step process through the direct benzimidazolization of paminobenzoic acid (PABA) and 3,3′-diaminobenzidine (DAB) in polyphosphoric acid. Poly(benzimidazole imide) (BI-PI) is then prepared through a two-step polycondensation process with oxydiphthalic anhydride (OPDA) followed by thermal imidization. The BI-PIs are woven into a membrane structure using an electrospinning technique and the membrane is hot-pressed for 30 min at 200 °C under a pressure of 50 kgf/cm^2^. Finally, the hot-pressed membrane is assembled into a supercapacitor as a separator, and its electrical performance is compared with that of a conventional cellulose separator.

## 2. Experimental Section

### 2.1. Materials

All of the reagents used in the present study were of ACS reagent grade and were used without any purification unless otherwise specified. 4-aminobenzoic acid (PABA) and 3,3′-diaminobenzidine (DAB) were acquired from Sigma-Aldrich (St. Louis, MO, USA). Polyphosphoric acid (PPA, 84 wt.%), phosphorus (V) oxide (P2O5), *N*,*N*-dimethylacetamide (DMAc) and dimethyl sulfoxide (DMSO) were acquired from Acros Organics (Thermo Fisher Scientific, Waltham, MA, USA). 4,4′-oxydiphthalic anhydride (ODPA) was acquired from Alfa Aesar, Thermo Fisher Scientific Co. (Waltham, MA, USA). Sodium bicarbonate and methanol were acquired from Duksan Pure Chemicals Co. (Ansan, South Korea). The *N,N*-dimethylacetamide (DMAc) and dimethyl sulfoxide (DMSO) were stirred in the presence of P2O5 overnight and then distilled under reduced pressure. 

### 2.2. Synthesis of 6,6′-bis[2-(4-Aminophenyl)Benzimidazole], BAPBI

PPA (40.0 g) and P_2_O_5_ (5.0 g) were mixed in a 100 mL completely dried reaction flask equipped with a mechanical stirrer (RCT digital, IKA^®^-Werke GmbH & CO. KG, Staufen, Germany) and a nitrogen inlet to form a thick paste. The mixture was stirred and heated at 150 °C until the P_2_O_5_ was completely dissolved and was then allowed to cool to room temperature. 3,3′-diaminobenzidine (2.1427 g, 10 mmol) and 4-aminobenzoic acid (3.0171 g, 22 mmol) were added to the flask and stirred for 12 h at 200 °C. After cooling to room temperature, the product was poured into an ice-cold 10 wt.% aqueous solution of sodium carbonate and was stirred rapidly to form a dark green precipitate. The solid precipitate was collected, washed with water and then dried. The dried precipitate was dissolved with methanol, and the filtrate was collected and vacuum dried to remove excess methanol and obtain yellow product BAPBI (3.79 g, 90%).

### 2.3. Fabrication of BI-PI Membrane

**Figure 1:**
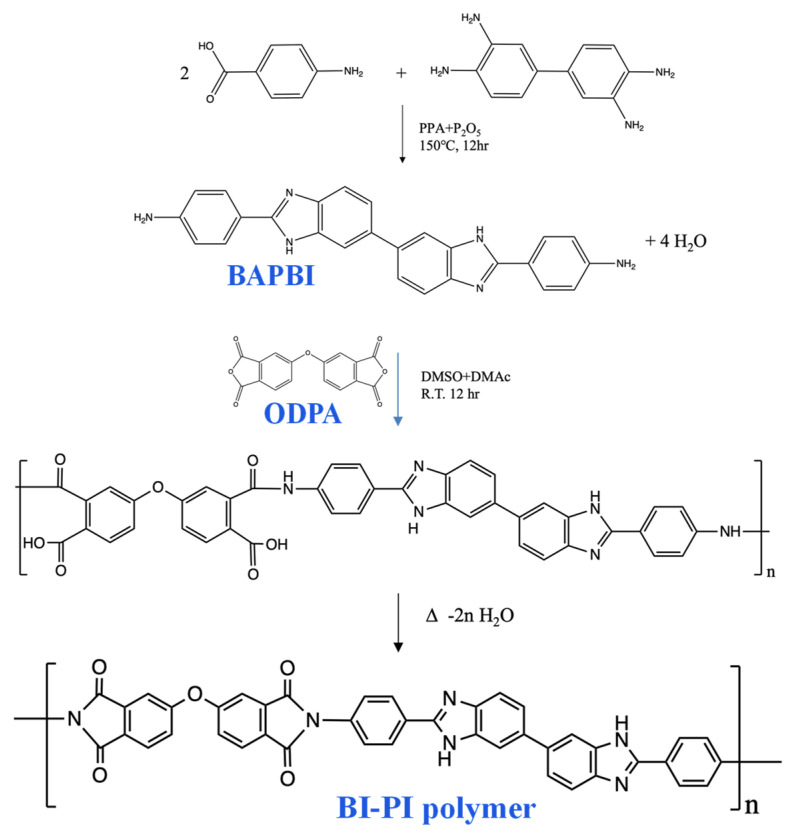
Synthesis of BI-PI polymer containing BAPBI.

BI-PI was synthesized using the two-step method shown in Figure 1. In a typical process, BAPBI (1.8758 g, 4.5 mmol) and ODPA (1.3980 g, 4.5 mmol) were mixed in 17.5 mL anhydrous DMSO and DMAc (volume ratio = 1:1). The mixture was stirred for 12 h at room temperature to obtain a homogenous viscous PAA solution with a solid content of 15.4 wt.%. Electrospinning was then performed using a syringe with a spinneret having an inside diameter of 0.686 mm (19 G) and an applied voltage of 20 kV. The PAA solution was fed at a speed of 0.01 mL/min with a distance of 20 cm between the tip of the needle and the collector. The PAA nanofiber-based nonwoven fabric was thermally imidized by a successive heating program (120, 200, 250, 300 °C for 2 h each and 350, 400 °C for 1 h each) in an Ar-condition muffle. Finally, the imidized BI-PI nonwoven fabric was hot-pressed for 30 min at 200 °C under a pressure of 50 kgf /cm^2^.

### 2.4. Characterization

Fourier transform infrared (FT-IR) spectra were obtained on a Thermo-iS50 spectrometer (Waltham, MA, USA) over the range of 400–4000 cm^−1^ with a 2 cm^−1^ spectral resolution. ^1^H-NMR and ^1^^3^C-NMR spectra were additionally obtained at 500 MHz using a Bruker AV-500 spectrometer (Bruker BioSpin, Rheinstetten, Germany) with DMSO-*d_6_* as the solvent. The surface morphologies and fiber diameters of the unpressed and hot-pressed BI-PI polymer membranes were examined by scanning electron microscopy (SEM, JSM-6701F, JEOL Ltd., Tokyo, Japan). Thermogravimetric analyses (TGA, Perkin Elmer TGA 4000, Washington, MA, USA) were conducted over the temperature range of room temperature to 800 °C at a heating rate of 10 °C /min under a dry nitrogen flow. The mechanical properties of the unpressed and hot-pressed BI-PI membranes were measured on a universal testing machine ((model 3365 with Bluehill software, Instron, Norwood, MA, USA) using rectangular specimens with a size of 150 × 5 mm and a stretching rate of 12.5 mm/min. Dynamic mechanical analyses (DMA, TA Q 800, TA Instruments, New Castle, DE, USA) were conducted in a nitrogen environment with a load frequency of 1 Hz and a heating rate of 5 °C/min over the temperature range of 100 to 450 °C. Finally, the pore-size distributions and porosities of the unpressed and hot-pressed membranes were measured by a mercury porosimeter (Micromeritics, AutoPore IV 9520, Norcross, GA, USA).

### 2.5. Electrochemistry Test

Multiporous carbon powder (MPC, Brunauer–Emmett–Teller surface area: 1500 m^2^/g) was derived by the KOH activation of water chestnut shell (WCS) at 800 °C, as described in a previous study by the present group [28]. Active materials (MPC, 92.5 wt.%) and polyvinylidene fluoride binder (7.5 wt.%) were thoroughly mixed to produce a slurry, and the slurry was coated on aluminum foils to produce test electrodes. A supercapacitor coin cell was constructed consisting of two facing electrodes sandwiching a hot-pressed BI-PI membrane (diameter 17 mm). Cyclic voltammetry (CV) measurements were conducted at voltages of −2.5 to 2.5 V using sweep rates ranging from 2 to 200 mV/s and 1.0 M TEABF_4_ in a propylene carbonate (PC) solution as the electrolyte. Electrochemical impedance spectroscopy (EIS) measurements were additionally conducted under open-circuit voltage conditions (0 V) using a frequency range of 1 mHz to 100 kHz and a 5 mV amplitude. The CV and EIS measurements were both obtained on an electrochemical workstation (CHI 680, CH Instruments, Inc., Austin, TX, USA). For comparison purposes, CV and EIS measurements were additionally obtained for a commercial CR-2032 coin cell with a cellulose membrane with a thickness of 30 μm (TF-4030, Nippon Kodoshi Corporation, Kochi, Japan). Coin cells were assembled in an argon-filled glove box which the oxygen and moisture content below 1.0 ppm.

## 3. Results and Discussions

### 3.1. Preparation and Characterization of BI-PI Polymer

**Figure 2:**
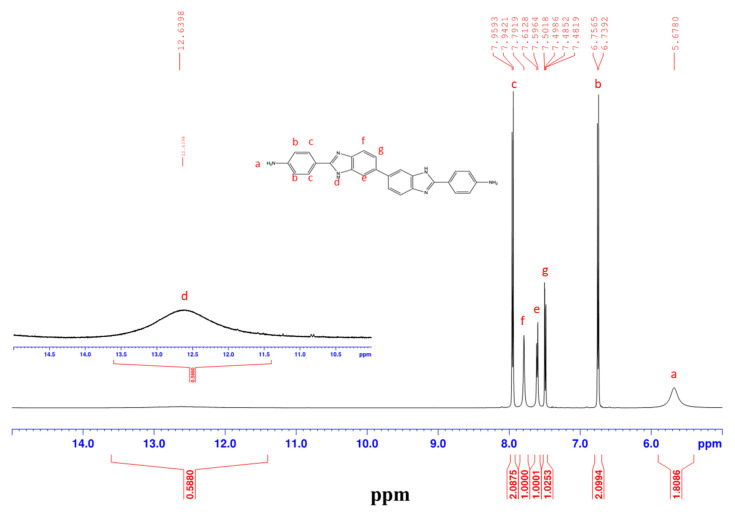
^1^H NMR spectra of BAPBI monomer in DMSO-*d*6.

A symmetric benzimidazole-containing diamine monomer (BAPBI) was synthesized by the direct benzimidazolization of paminobenzoic acid (PABA) and 3,3′-diaminobenzidine (DAB) in polyphosphoric acid. The structure of the synthesized BAPBI was confirmed by an inspection of the ^1^H NMR (Figure 2) and ^13^C NMR (Figure S1) spectra, in which the chemical shifts and signal integration ratios were found to be consistent with those of the BAPBI compound [13]. Berrada et al. [13] and Liu et al. [14] also synthesized symmetric benzimidazole-containing diamines. However, the synthesis routes in [13,14] were complex, and the corresponding yields were just 40 and 67%, respectively. By contrast, the BAPBI synthesis route adopted in the present study is a simple one-pot process with a high yield of 90%.

**Figure 3:**
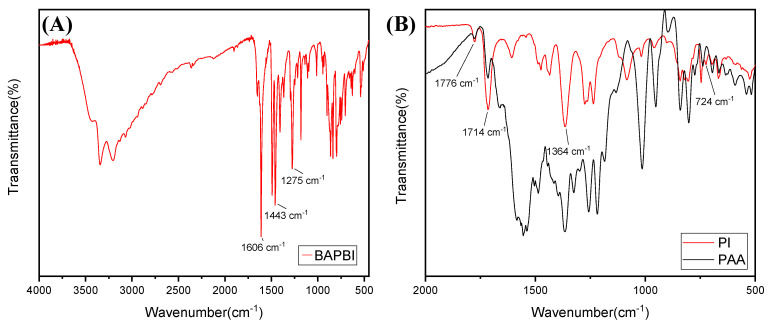
FT-IR spectra of (**A**) BAPBI monomer, (**B**) PAA polymer and BI-PI polymer.

The BAPBI was used to prepare the BI-PI polymer through a polycondensation process in the presence of an ODPA. In particular, the PAA was prepared by a solution polymerization process in DMSO and DMAC (volume ratio = 1:1), and a PAA nanofiber-based nonwoven fabric was then obtained by thermal imidization, using a programmed heating procedure (Section 2.3). The FT-IR spectra of the BAPBI (Figure 3A) showed absorption bands at 1275 cm^−1^ (imidazole ring breathing), 1606 cm^−1^ (ring vibration of conjugation between benzene and imidazole rings) and 1443 cm^−1^ (in-plane vibration of 2,6-disubstituted benzimidazole ring), where all of these bands are attributed to the benzimidazole group. Absorption bands were also observed in the FT-IR spectra of the BI-PI polymer (Figure 3B) at 1776 cm^−1^ (C=O asymmetric stretching), 1714 cm^−1^ (C=O symmetric stretching), 1364 cm^−1^ (C–N stretching) and 724 cm^−1^ (imide ring deformation), corresponding to characteristic imide bands. Overall, the NMR and FT-IR data confirm the successful synthesis of the BI-PI polymer through the adopted synthesis route.

**Figure 4:**
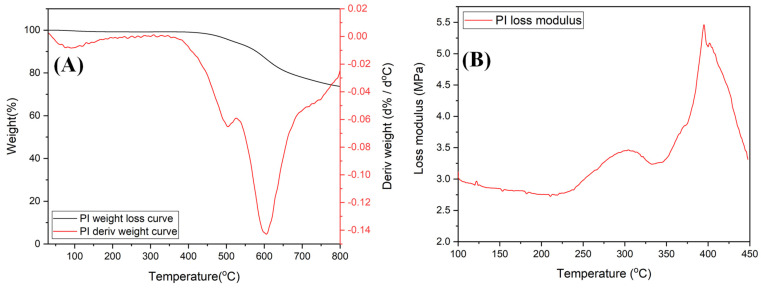
(**A**) TGA and (**B**) DMA curves of BI-PI membrane.

The thermal properties of the BI-PI polymer were analyzed by a TGA and DMA, as shown in Figure 4A,B, respectively. As shown in Figure 4A, a small weight loss occurred at around 100 °C while a large weight loss occurred at temperatures greater than 500 °C. The initial weight loss is the result primarily of the loss of water through evaporation, while the latter loss is attributed to the thermal decomposition of the polymer backbone [13,14,15]. The 5 wt.% weight-loss temperature is equal to 527 °C, and the amount of carbonized residue of the polymer material at a temperature of 800 °C is 73.7%. It is noted that both values are consistent with those previously reported for benzimidazole-containing PIs [14,15]. The weight-loss temperatures of the present BI-PI polymer are remarkably high as a result of their composition and structure, which consists of benzimidazole segments and high-temperature-resistant imide units. However, previous studies have suggested that the high-temperature resistance of benzimidazole-containing polymers may also stem in part from their rigid-rod structures, which suppress morphological changes under high-temperature conditions [26].

As shown in Figure 4B, the BI-PI polymer has two glass transition temperatures of Tg = 303 and 395 °C, respectively. The existence of two Tg values arises due to the presence of both soft-chain segments of flexible ether bonds and hard-chain segments of imide and benzimidazole in the polymer backbone. These soft-chain segments rotate easily, whereas the motion of the hard-chain segments is more constrained. The higher Tg value at 395 °C is caused by the large number of aromatic rings and intermolecular hydrogen bonds of imidazole in the main chain [14]. The XRD pattern presented in Figure S2 confirms that the BI-PI polymer has an amorphous structure.

### 3.2. Preparation and Characterization of Electrospun BI-PI Membrane

**Figure 5:**
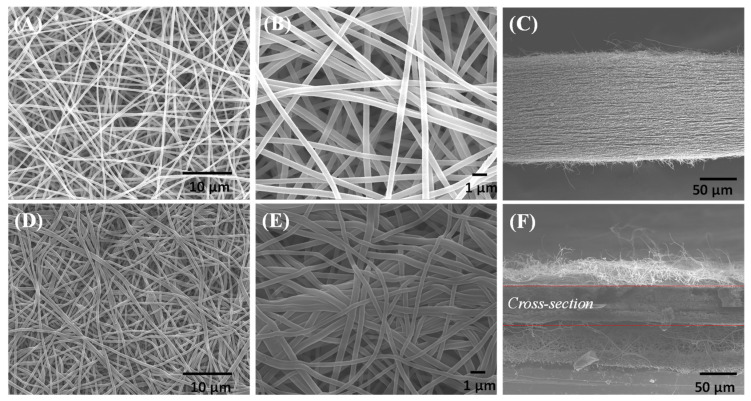
SEM images of (**A**–**C**) raw BI-PI membrane and (**D**–**F**) hot-pressed BI-PI membrane.

**Figure 6:**
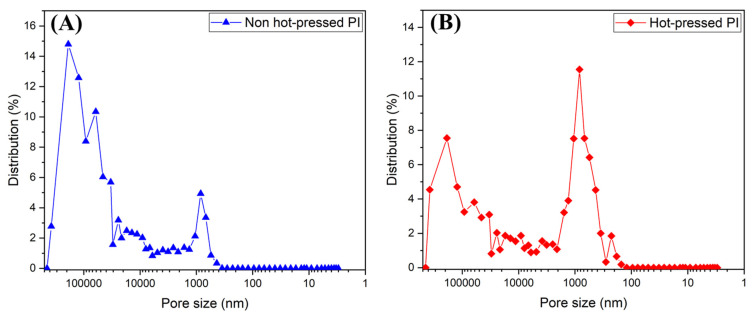
Pore size–distribution curves of (**A**) raw BI-PI membrane and (**B**) hot-pressed BI-PI membrane.

The BI-PI polymer was prepared into a high-porosity membrane by electrospinning, as described in Section 2.3. The membrane was then hot pressed at a temperature of 200 °C for 30 min under a pressure of 50 kgf/cm^2^. The morphologies, porosities and mechanical properties of the two membranes were then evaluated and compared. Figure 5A,B,D,E present SEM images of the surface morphologies of the raw and hot-pressed membranes, respectively. For both membranes, the pore diameter distributions of cross-stacked pores are reasonably uniform, and the average pore diameter size is around 400 nm. However, the BI-PI fibers in the hot-pressed membrane show signs of partial melting. The cross-sectional SEM images presented in Figure 5C,E show that the unpressed and hot-pressed membranes have thicknesses of approximately 150 and 50 μm, respectively. In other words, the hot-press process leads to a densification of the membrane structure and reduces the membrane porosity from 91% (unpressed membrane) to 73% (hot-pressed membrane), as determined by mercury porosimeter. Figure 6 shows the pore-size distribution analysis results for the two membranes. As shown in Figure 6A, the unpressed BI-PI membrane consists mainly of large pores with a size of 10,000 nm and a lesser quantity of small pores with a size of 1000 nm, where the different pore sizes correspond to the voids stacked between the fiber layers of the membrane and the cross-stacked pores between the fibers, respectively (see Figure 6A,B). Figure 6B shows that the hot-press process reduces the number of large pores in the membrane, with the result that the membrane structure is dominated by small cross-stacked pores with a size of approximately 1000 nm between the fibers. The more uniform pore-size distribution in the hot-pressed membrane leads to a more uniform ion diffusion through the membrane. Consequently, concentration gradients are less readily formed, and the electrochemical performance of the membrane is correspondingly improved [28,29,30]. For comparison purposes, Figure S3 presents SEM images of the surface morphology of the commercial TF-4030 separator. It is seen that that the separator has a broader pore-size distribution than the hot-pressed BI-PI membrane and a generally smaller pore size. Consequently, the electrochemical stability of the membrane with a uniform pore size in supercapacitor applications will be greatly improved.

**Figure 7:**
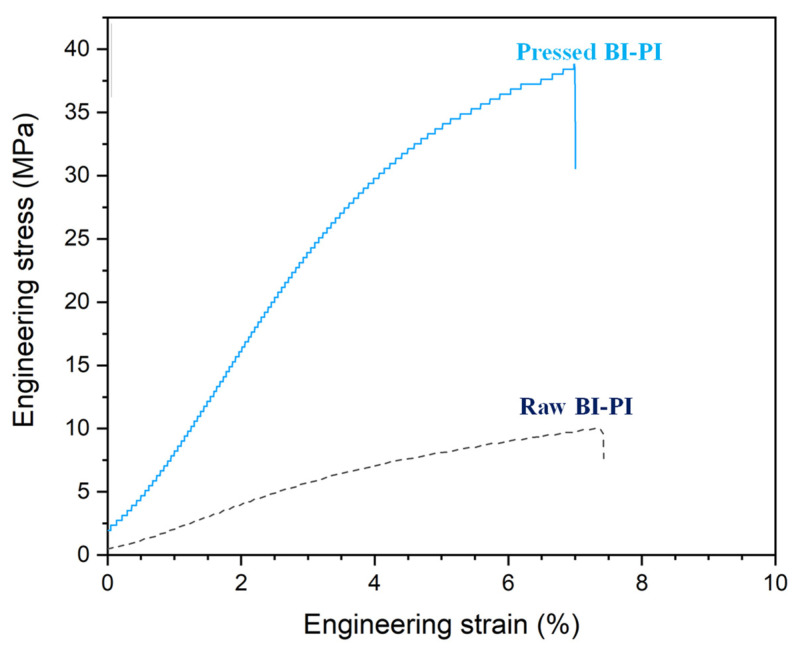
Stress–strain curves of raw BI-PI membrane and hot-pressed BI-PI membrane.

Figure 7 shows the stress–strain curves of the unpressed and hot-pressed BI-PI membranes, respectively. The mechanical properties of the two membranes are summarized in Table 1. As shown, the raw membrane has a tensile modulus of 203 MPa and a tensile strength of 9.9 MPa. Previous studies have reported that the extensive delocalization of π electrons in benzimidazoles generates an array of favorable characteristics, including outstanding mechanical and dielectric properties [23]. Furthermore, the prolonged and gentle heating effect produced in the imidization process results in a relaxation of the polymer chains in the BI-PI membrane and a more stable conformation, which improve the thermal stability [16,17,18]. Finally, the hydrogen bondings formed between the macromolecular chains of the BI-PI via the imidazole ring of the BAPBI and the carbonyl groups also contribute to a high tensile strength and modulus of the membrane [13]. However, as shown in Table 1, the hot-press process results in a significant improvement in both the tensile modulus (783 MPa) and the tensile strength (34.8 MPa) of the membrane due to the densification effect shown in Figure 5F (SEM image). Consequently, the durability of the membrane in harsh environments is greatly improved.

The thermal stability of separators is an important safety concern in supercapacitor applications. Accordingly, the thermal dimensional stability of the present hot-pressed BI-PI membrane was investigated in an oven at temperatures of 80, 120, 150 and 200 °C for 30 min. For comparison purposes, a commercial PP/PE membrane (Celgard H1612, AL, USA) was also evaluated under the same conditions. Figure S4 presents experimental images of the two membranes after each heating period. It can be seen that the BI-PI membrane undergoes virtually no change in dimensions in the different tests. By contrast, the PE/PP separator shows significant curling and shrinkage after heating at 120 °C and melts almost completely at 150 °C. The improved thermal stability of the BI-PI membrane can be attributed to the high melting temperature of the PI components of its structure [17,18,19].

### 3.3. Supercapacitor Application of BI-PI Separator

**Figure 8:**
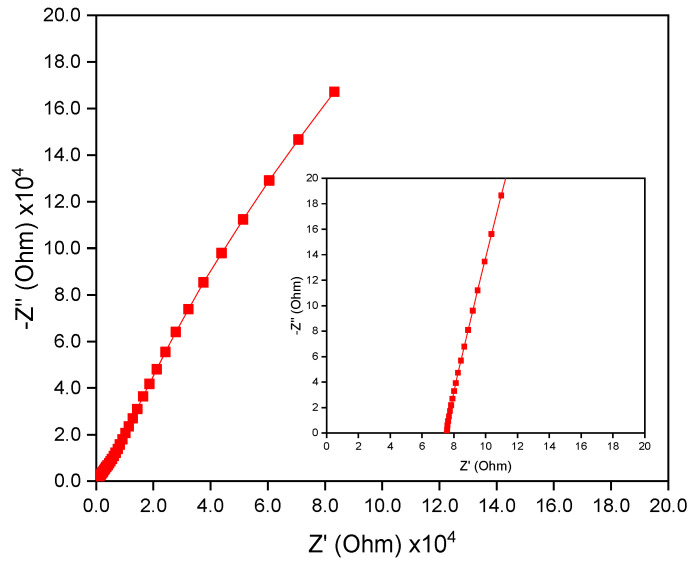
Nyquist impedance plot of hot-pressed BI-PI membrane. The inset shows a magnified view of the high-frequency region of the impedance spectrum.

Figure 8 shows the Nyquist plot of the hot-pressed BI-PI membrane when assembled in a stainless-steel cell. The plot has a high slope, which indicates that the separator has a low impendence toward the electrode surface. The ionic conductivity (ó) of the membrane can be evaluated as

ó = *R*_I_^−1^ *A*^−1^ *d*, (1)

where *R*_I_ is the intercept of the Nyquist plot on the real axis, *A* is the geometric area of the electrolyte/electrode interface and *d* is the distance between the two electrodes (50 μm) [31]. The ionic conductivity of the membrane was found to be 2.02 mS/cm. The high ionic conductivity can be attributed to the intimate contact of the BI-PI separator with the electrode surface and the high degree of ion dissociation caused by the strong affinity between the ether linkage and the solvent molecules, which promotes a swelling of the polymeric framework [13]. In addition, the BI-PI membrane showed an excellent electrolyte wettability, with a contact angle close to 0°, as shown in Figure S5. In summary, the high conductivity of the membrane is achieved.

**Figure 9:**
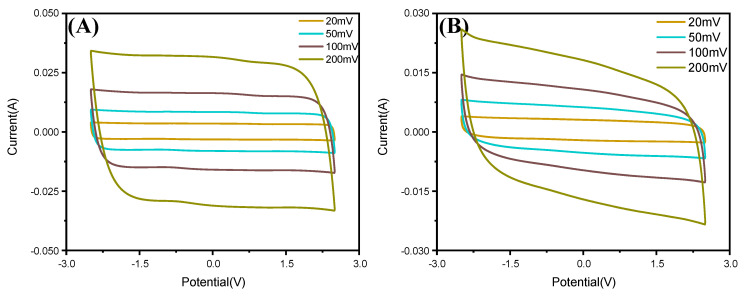
CV plots of symmetric two-electrode capacitors assembled with the (**A**) hot-pressed BI-PI separator and (**B**) commercial TF-4030 cellulose separator.

**Figure 10:**
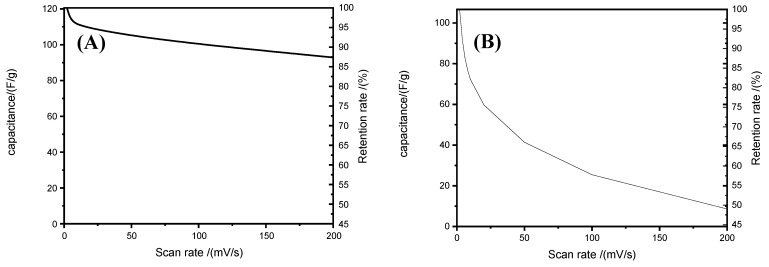
Specific capacitance and capacitance retention vs. scan rate for (**A**) hot-pressed BI-PI capacitor and (**B**) commercial TF-4030 cellulose capacitor.

Figure 9 show the CV potential scanning curves for supercapacitors assembled using the proposed BI-PI membrane (Figure 9A) and a commercial cellulose separator (Figure 9B), respectively. At a low scan rate of 2 mV/s, the BI-PI supercapacitor showed a specific capacitance value of 121 F/g. By contrast, the cellulose supercapacitor had a specific capacitance of 107 F/g. Furthermore, the BI-PI supercapacitor exhibited a rectangular voltammogram typical of that of an ideal double-layer capacitor at both low and high scan rates. However, the voltammograms for the cellulose separator system showed a more distorted rectangular shape, indicating the existence of ohmic resistance on the ion motion through the membrane. The distortion effect was particularly pronounced at higher scan rates. This effect may be attributed to the gradual intrusion of the ohmic resistance for ion motion in the pores, or even in the bulk phase, at higher scan rates [28,32,33]. In particular, the presence of ohmic-resistance results in a potential gradient, which decreases the capacitance of the electrodes at high charge-storage rates. Figure 10 shows the capacitance retention of the two supercapacitors with the BI-PI membrane and commercial cellulose separator, respectively. It is evident that the BI-PI supercapacitor has both a higher specific capacitance than the commercial supercapacitor and a higher capacitance retention (i.e., 77 vs. 49%).

**Figure 11:**
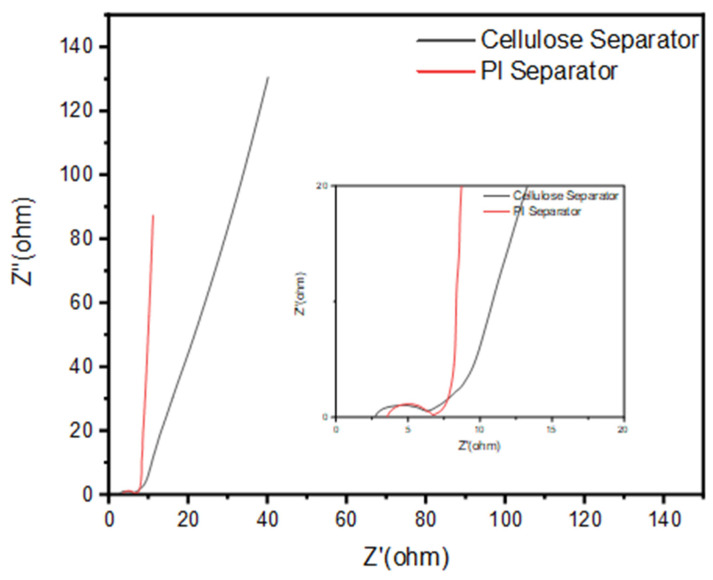
Nyquist impedance plots of the capacitor using BI-PI separator and cellulose separator, respectively, over frequency range of 100 kHz to 10 mHz and applied potential of 0 V. The inset shows a magnified view of the high-frequency region of the impedance spectra.

Figure 11 shows the Nyquist-type impedance spectra of the BI-PI supercapacitor and cellulose supercapacitor, respectively. The real-part resistance in the high-frequency region comprises both the equivalent series resistance (R_s_) of the supercapacitor (attributed to the resistance of the electrolyte and the separator) and the interfacial resistance (R_ct_) of the supercapacitor [15]. The full-scale spectra in Figure 11 have the form of almost vertical lines, indicating that both cells have a good capacitive performance. In general, a higher slope of the Nyquist plot indicates a lower impedance of ion conduction within the system [15]. Thus, the results indicate that the BI-PI supercapacitor has a lower Rs resistance than the cellulose supercapacitor. The interfacial resistance, R_ct_, of a supercapacitor is associated with the resistance created by ion migration in the carbon electrode and separator region, respectively [15]. The BI-PI capacitor and cellulose capacitor have R_ct_ values of 3.14 and 3.53 Ω, respectively, as determined by the impedance spectra. Overall, the results show that the BI-PI capacitor has both a small R_ct_ value and a low R_s_ resistance, and thus provides an extraordinarily fast charge−discharge performance.

## 4. Conclusions

This study has employed a simple two-step synthesis route to prepare the BI-PI from symmetrical rigid-rod benzimidazole-containing diamine monomers (BAPBI). An electrospinning technique has then been used to fabricate BI-PI membranes. The thermal, mechanical and electrical properties of the BI-PI membranes have been investigated in both a raw condition and a hot-pressed condition (30 min, 200 °C, 50 kgf/cm^2^). The characterization results have shown that the BI-PI membrane has good thermal stability with a 5 wt.% weight-loss temperature of 527 °C. The good thermal performance of the membrane has been attributed to the rigid-rod structure of the BI-PI and the presence of high-temperature-resistant imide units, respectively. The SEM observations have shown that the hot-press process reduces the number of large pores in the membrane and improves the uniformity of the pore-size distribution. Moreover, the hot-press process results in a significant densification of the membrane structure, and hence improves the tensile modulus and tensile strength from 203 and 9.9 MPa, respectively, in the raw condition to 783 and 34.8 MPa in the hot-pressed condition. In supercapacitor applications, the hot-pressed membrane has shown a specific capacitance of 121 F/g, which compares favorably with that of 107 F/g obtained for a supercapacitor with a commercial cellulose separator. Furthermore, the BI-PI supercapacitor has shown a capacitance retention rate around 1.57 times higher than that of the commercial cellulose supercapacitor (i.e., 77 vs. 49%, respectively) and has both a lower equivalent series resistance (R_s_) and a lower interfacial resistance (R_ct_). Overall, the results have shown that the hot-pressed BI-PI membrane has good thermal stability, superior mechanical properties and an outstanding electrochemical performance. It thus provides an ideal separator for supercapacitors and other clean-energy storage systems.

## Figures and Tables

**Table 1:** Mechanical properties of raw BI-PI and pressed BI-PI membranes.

	Thickness/μm	Modulus/MPa	Tensile Strength/MPa	Elongation/%
Raw BI-PI	150	202.9 ± 6.1	9.9 ± 1.5	7.8 ± 1.2
Pressed BI-PI	50	783 ± 50	35 ± 4	6.1 ± 0.6

## Data Availability

Not applicable.

## Supplementary Materials

The following supporting information can be downloaded at: https://www.mdpi.com/article/10.3390/membranes12100961/s1, Figure S1. 13C NMR spectra of BAPBI in DMSO-d6; Figure S2. XRD diagram of BI-PI polymer; Figure S3. SEM images of commercial cellulose separator (TF-4030); Figure S4. Thermal dimensional stability test results for BI-PI separator and Celgard H1612 separator.; Figure S5. Contact angle test results for hot-pressed BI-PI separator.
