# Supplementary material for: Electrospun Benzimidazole-Based Polyimide Membrane for Supercapacitor Applications
Source: Membranes (Basel). 2022 Sep 30;12(10):961. doi: 10.3390/membranes12100961 (PMC9607089; doi:10.3390/membranes12100961)
Supplement: Supplementary file 1 [file membranes-12-00961-s001.zip › membranes-1924909-supplementary.pdf]

membranes

MDPI

Article

Electrospun Benzimidazole-Based Polyimide Membrane for Supercapacitor Applications

Yu-Hsiang Lu [1], Yen-Zen Wang [1,*], Ming-Ying Tsai [2], Hong-Ping Lin [2] and Chun-Han Hsu [3,*]

[1] Department of Chemical and Materials Engineering, National Yunlin University of Science and Technology, No. 123, Sec. 3, University Road, Douliou 64002, Taiwan
[2] Department of Chemistry, National Cheng Kung University, No. 1 University Road, Tainan City 70101, Taiwan
[3] General Education Center, National Tainan Junior College of Nursing, No. 78, Sec. 2, Minzu Road, Tainan City 700, Taiwan
* Correspondence: wangzen@yuntech.edu.tw (Y.-Z.W.); chhsu@ntin.edu.tw (C.-H.H.)

Citation: Lu, Y.-H.; Wang, Y.-Z.; Tsai, M.-Y.; Lin, H.-P.; Hsu, C.-H. Electrospun Benzimidazole-Based Polyimide Membrane for Supercapacitor Applications. *Membranes* **2022**, *12*, 961. https://doi.org/10.3390/membranes12100961

Academic Editor: Scott M. Husson

Received: 3 September 2022
Accepted: 26 September 2022
Published: 30 September 2022

Publisher's Note: MDPI stays neutral with regard to jurisdictional claims in published maps and institutional affiliations.

Copyright: © 2022 by the authors. Licensee MDPI, Basel, Switzerland. This article is an open access article distributed under the terms and conditions of the Creative Commons Attribution (CC BY) license (https://creativecommons.org/licenses/by/4.0/).

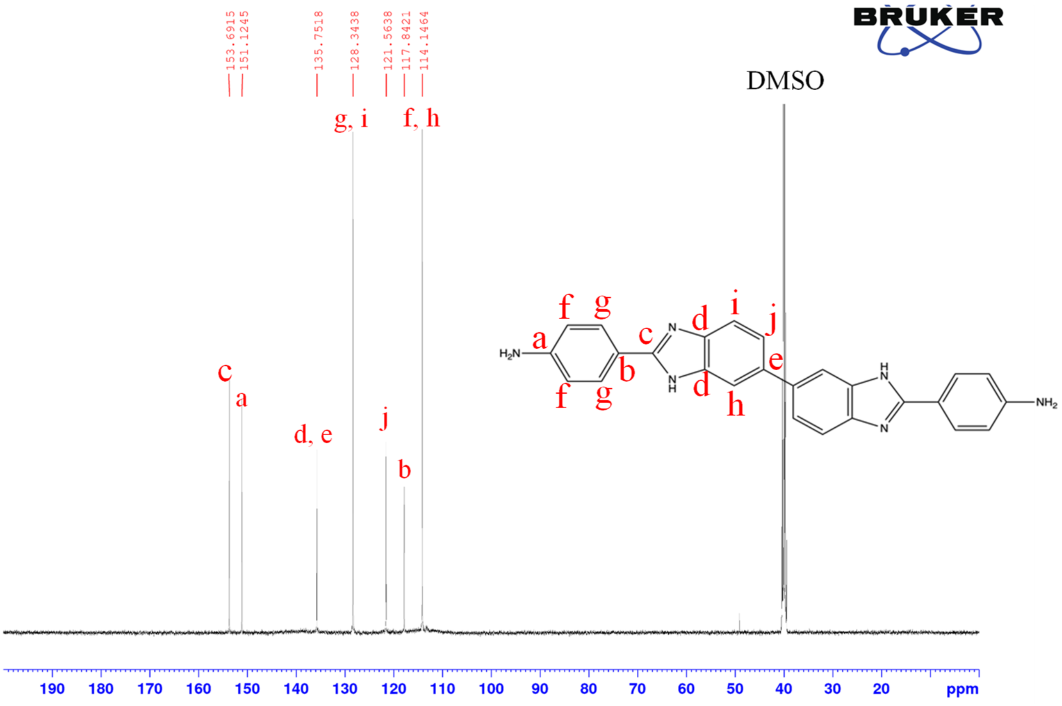

Figure S1. ^{13}C NMR spectra of BAPBI in DMSO-*d6*

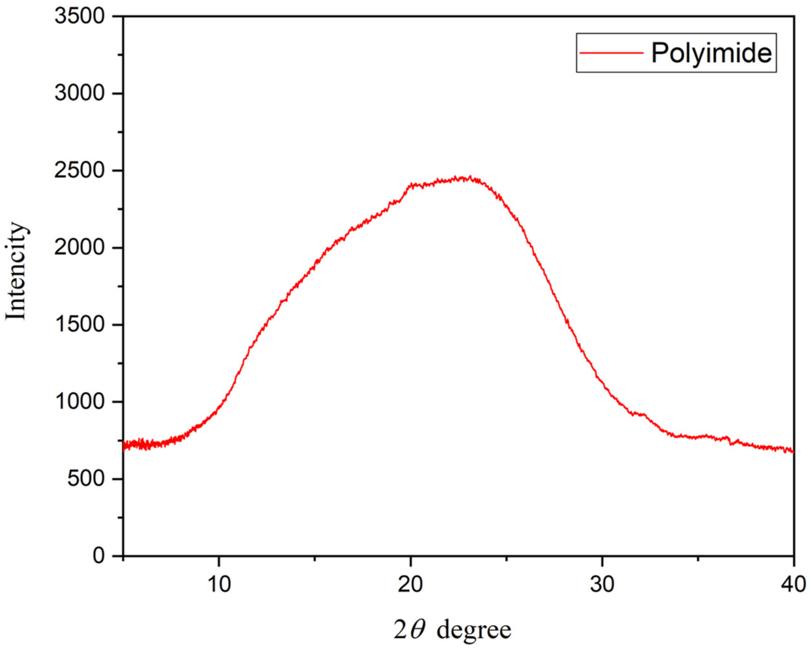

Figure S2. XRD diagram of BI-PI polymer.

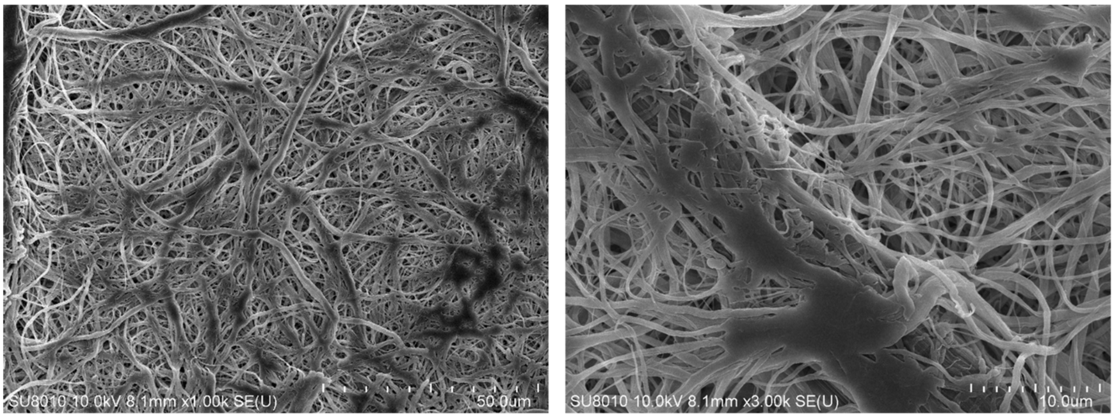

Figure S3. SEM images of commercial cellulose separator (TF-4030).

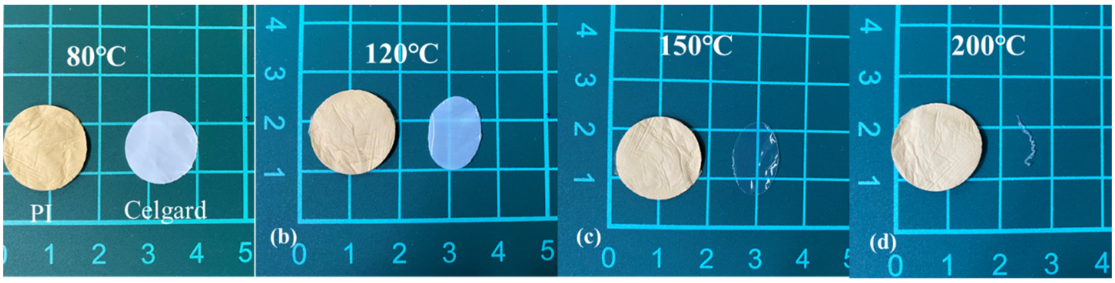

Figure S4. Thermal dimensional stability test results for BI-PI separator and Celgard H1612 separator.

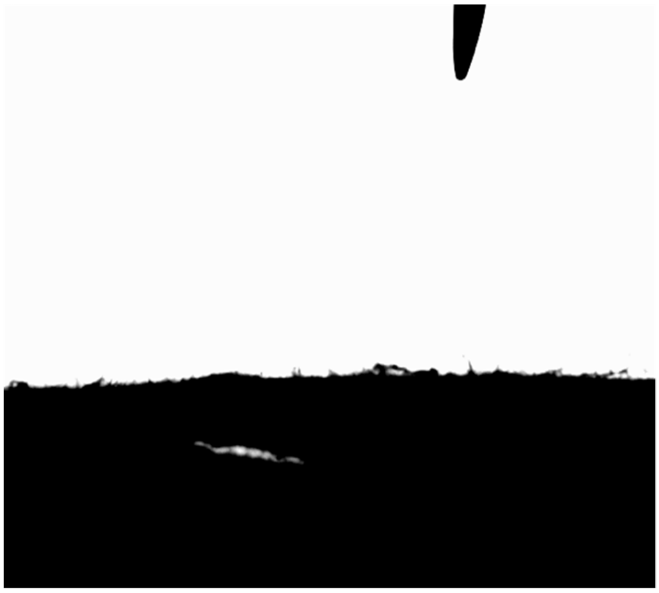

Figure S5. Contact angle test results for hot-pressed BI-PI separator.
